# Treatment Persistence in Migraine Prophylaxis Comparing CGRP Monoclonal Antibodies vs. High-/Low-Evidence Conventional Oral Preventives—A Comparative Real-World Evidence Study of Depersonalized Data of the German Pain e-Registry

**DOI:** 10.3390/jcm15051985

**Published:** 2026-03-05

**Authors:** Michael A. Überall, Philipp C. G. Müller-Schwefe, Michael A. Küster, Jan-Peter Jansen

**Affiliations:** 1 IFNAP—Private Institute of Neurological Sciences, O. Meany-MDPM GmbH, Nordostpark 51, 90411 Nürnberg, Germany; 2 Interdisciplinary Center for Pain & Palliative Care Medicine, Schillerplatz 8/1, 73033 Göppingen, Germany; philipp@mueller-schwefe.com; 3 Interdisciplinary Center for Pain & Palliative Care Medicine, 53177 Bonn, Germany; drkuester@gmx.de; 4 Pain Medicine Center Berlin, Schönhauser Allee 172 a, 10435 Berlin, Germany; gf@schmerzmedizin.berlin

**Keywords:** migraine, prevention, CGRP, treatment persistence, real-world evidence, German Pain e-Registry

## Abstract

**Background/Objectives**: Real-world persistence of traditional oral migraine preventive medications is low in routine care. Prior large claims-based analyses demonstrated early discontinuation of oral prophylaxis, but such datasets neither included modern preventive options such as monoclonal antibodies (mAB) against calcitonin-gene-related peptide (CGRP), nor were able to capture clinically validated reasons for discontinuation. The primary aim was to compare real-world treatment persistence and discontinuation reasons due to adverse drug reactions (ADRs) or insufficient efficacy among three preventive therapy classes: subcutaneous CGRP mAB and oral high- (HEVP) and low-evidence preventive medications (LEVP). A secondary aim was to examine persistence patterns of individual substances within the HEVP cohort. **Methods**: This exploratory observational study used depersonalized real-world data from the German Pain e-Registry (GPeR), a national multicenter clinical registry. Persistence trajectories were evaluated over six months, together with cumulative proportions of ADR-related and inefficacy-related discontinuations. Pairwise comparisons across the three cohorts based on chi-square analyses, odds ratios, relative risks, effect sizes, and numbers needed to harm. **Results**: At six months, persistence was highest for CGRP monoclonal antibodies at 89.4%, compared with 43.0% for LEVP and 34.0% for HEVP (all *p* < 0.001). ADR-related discontinuation occurred in 7.0% with CGRP vs. 35.7/44.5% with LEVP/HEVP, and discontinuations due to insufficient efficacy occurred in 3.6% with CGRP vs. 21.3/21.5% with LEVP/HEVP, without influence of sex or migraine frequency. Substance-level analysis within HEVP showed the steepest early attrition for tricyclic antidepressants, followed by beta-blockers, with comparatively more favorable though still suboptimal persistence for topiramate and flunarizine. **Conclusions**: Real-world treatment persistence is markedly higher with CGRP mAB than with HEVP/LEVP. Oral preventives show high discontinuation rates due to both ADR and insufficient efficacy, indicating substantial limitations in real-world applicability. These findings highlight the clinical relevance of a modern mechanism-based migraine prevention with CGRP mAB.

## 1. Background and Rationale

Migraine is a highly prevalent neurological disorder associated with substantial disability and reduced quality of life, particularly in individuals with frequent attacks or chronic migraine, for whom preventive therapy is a central component of disease management [1,2]. Over the past decades, oral migraine preventive medications, including beta-blockers, tricyclic antidepressants, and anticonvulsants such as topiramate and valproic acid, as well as calcium channel blockers like flunarizine, have formed the foundation of prophylactic care and were recommended by evidence-based guidelines [3]. Nevertheless, real-world adherence and persistence to these conventional preventives (i.e., the continuous use of the preventive therapy without interruption over a defined observation period, reflecting an explicit physician–patient decision rather than prescription refill surrogates) remain consistently low. A landmark claims-based analysis by Hepp and colleagues reported that among chronic migraine patients initiating oral prophylaxis, only about one-quarter were still persistent at 6 months and only about 14% at 12 months, with the greatest attrition occurring within the first one to two months of therapy [4]. This pattern has been confirmed across multiple observational settings and systematically summarized in real-world evidence reviews showing low persistence and early discontinuation irrespective of oral preventive class [5,6].

Despite the importance of this US claims analysis, several limitations make a contemporary registry-based evaluation necessary. First, claims data document prescription fills but cannot determine clinically validated reasons for discontinuation. They infer persistence from refill gaps rather than from patient-decided and physician-verified therapy termination and therefore cannot distinguish whether cessation results from adverse reactions or insufficient effects. Real-world registry studies are uniquely suited to address this gap because they record, among others, discontinuation reasons [6]. Second, the timeframe of the Hepp dataset (2008–2012) predates the introduction of monoclonal antibodies targeting the calcitonin gene-related peptide (CGRP) or its receptor. These agents represent a fundamentally different, mechanism-driven preventive strategy whose real-world persistence and tolerability are crucial to evaluate [7,8]. Third, the US-claims analysis did not incorporate oral preventive therapies of lower evidence level (“low-evidence preventives”, LEVP), which remain in routine use for some patients in everyday clinical care and require real-world comparison with both high-evidence preventives (HEVPs) and modern biologics [6]. Fourth, the population examined was shaped by US insurance structures, referral pathways, and prescribing practices, limiting the generalizability to healthcare systems in the rest of the world.

The German Pain e-Registry (GPeR) provides a nationwide multicenter specialist-care dataset in which migraine diagnoses are physician-confirmed and preventive treatments are documented longitudinally together with explicit reasons for discontinuation. This enables a clinically grounded assessment of therapy survival for both conventional and modern preventive strategies. Contemporary international real-world studies already indicate that CGRP monoclonal antibodies achieve higher persistence and drug survival than traditional preventives, although absolute continuation rates vary depending on healthcare systems and analytic conventions [7,8,9,10,11]. For example, nationwide registry data from Norway, multicenter cohorts from Spain and other European countries, and large US observational datasets consistently report higher drug survival and lower discontinuation rates for CGRP-targeted therapies compared with conventional oral preventives. The present findings therefore align with, and extend, this growing international real-world evidence base by adding clinically validated discontinuation reasons and substance-level analyses within a large German cohort. Against this background, the present analysis was designed to provide a benchmark comparing CGRP monoclonal antibodies with both high-evidence and low-evidence oral preventives, and to describe substance-level persistence within high-evidence oral therapies. The study focused specifically on treatment persistence and on the two dominant clinical drivers of early preventive therapy failure, namely adverse drug reactions (ADRs) and insufficient efficacy, because these reasons capture the central patient- and physician-related determinants of real-world treatment acceptability [4,5,12].

## 2. Study Aims

The primary aim of this study was to evaluate and compare real-world treatment persistence, adverse drug reaction-related discontinuation, and discontinuation due to insufficient efficacy among patients receiving subcutaneous CGRP mAB as well as oral HEVP and LEVP. The secondary aim was to examine substance-specific persistence patterns within the HEVP cohort, including beta-blockers, tricyclic antidepressants, flunarizine, valproic acid, and topiramate.

## 3. Methods

The present investigation was conducted as an exploratory observational, registry-based cohort study using depersonalized 6-month treatment data from the GPeR, a large, multicenter, longitudinal clinical registry capturing routine care in specialized pain centers across Germany. The registry documents sociodemographic variables, diagnoses, treatment histories, therapeutic decisions, and detailed reasons for therapy modifications or discontinuations. The GPeR was established in January 2015, and for the present analysis, treatment episodes documented between January 2015 and December 2024 were evaluated, corresponding to a period in which CGRP monoclonal antibodies were already available in routine care.

In the German healthcare system, patients with frequent or refractory migraine are typically first managed by general practitioners and office-based neurologists, while referral to specialized pain or headache centers is common when multiple preventive treatments have failed or were not tolerated. In accordance with national reimbursement regulations, CGRP monoclonal antibodies are generally prescribed only after documented failure or intolerance of at least one conventional oral preventive therapy. Follow-up visits in routine care and in the registry usually occur at intervals of ~12 weeks after treatment initiation and adjustments.

Data are collected prospectively by treating physicians and patients as part of standardized documentation requirements within participating practices, thereby ensuring clinical accuracy and minimizing recall bias. For the purpose of this analysis, only patients with a physician-confirmed diagnosis of migraine receiving preventive medication were included. Because the registry captures clinically validated diagnoses, rather than administrative codes as in claims datasets, the study population reflects real-world clinical decision-making in daily practice.

### 3.1. Study Design and Cohort Definition

Three treatment cohorts were defined based on the preventive therapy initiated or continued at baseline: subcutaneous CGRP monoclonal antibodies (CGRP cohort), high-evidence preventive medications (HEVP cohort), and low-evidence preventive medications (LEVP cohort). The CGRP cohort comprised all patients treated with any subcutaneous CGRP monoclonal antibody, aggregated into a unified group because this analysis focused on treatment persistence rather than comparative efficacy between individual biologics. The HEVP cohort included oral preventive medications with established guideline-level evidence for migraine prophylaxis: beta-blockers, tricyclic antidepressants, flunarizine, valproic acid, and topiramate. Within this HEVP cohort, substance-specific subgroups were retained, allowing secondary analyses of substance-level persistence. The LEVP cohort comprised oral preventive therapies widely used in clinical practice but lacking strong evidence from randomized controlled trials, such as other anticonvulsants (e.g., gabapentin and levetiracetam), specific antidepressants (such as mirtazapine, etc.), angiotensin II type 1 (AT1) receptor blockers (e.g., candesartan, etc.), angiotensin-converting-enzyme (ACE) inhibitors (such as captopril, etc.), and riboflavin or coenzyme Q10, etc. During the evaluation period, no patients in the GPeR received oral preventive gepants (e.g., atogepant or rimegepant), as these agents were not yet approved or routinely available in Germany. Patients could only be included once per evaluation period. Baseline preventive therapy defined their cohort membership, and the date of first use defined the start of the 6-month evaluation period. Onabotulinumtoxin A was not included in the present analysis, as the study focused exclusively on pharmacological oral and subcutaneous preventive agents documented in the registry, and injection procedures with distinct administration intervals and reimbursement pathways would have required a separate analytic framework.

As this study was explicitly designed to evaluate naturalistic treatment trajectories rather than impose a controlled trial framework, no exclusion criteria were imposed regarding the number of prior preventive therapies, comorbidities, or concomitant treatments, to preserve the real-world representativeness of the analysis.

### 3.2. Evaluation Period and Endpoints

The evaluation window spanned 182 days (corresponding to 26 weeks or roughly six months), reflecting the clinically relevant timeframe for evaluating treatment efficiency, persistence, and discontinuation. For each patient, the baseline date was defined as the date on which the preventive therapy under investigation was initiated or, if already initiated, first documented within the registry period analyzed.

Importantly, the present analysis focused solely on treatment retention and the two principal reasons for early discontinuation, namely ADR and insufficient therapeutic efficacy. These two parameters were chosen because they represent the dominant clinical drivers of real-world therapy termination and thereby offer a direct measure of the combined effects of tolerability, therapeutic benefit, and patient acceptance. Unlike broader outcomes such as headache frequency or functional impairment, which require systematic longitudinal symptom assessment, early discontinuation constitutes a “hard” clinical endpoint resulting from the explicit physician–patient decision to stop a therapy. As such, it embodies an integrated assessment of clinical effectiveness and tolerability as perceived in daily practice.

Baseline demographic and clinical characteristics—including sex, age, migraine duration, and migraine type (episodic vs. chronic)—were additionally extracted, summarized descriptively, and used as external control parameters to provide information on their influence on treatment discontinuations.

### 3.3. Documentation of Treatment Persistence

Treatment persistence was quantified as the proportion of patients who remained on the index preventive therapy without interruption until the end of the 182-day evaluation period. Persistence trajectories were derived from daily GPeR data and used to calculate Kaplan–Meier survival curves, which capture the cumulative proportion of patients still receiving therapy. In addition, discontinuation rates at one, two, three, and six months were calculated with respect to the reasons given. Because the registry records physician-confirmed treatment continuation with reference to patient decision, persistence reflects actual therapeutic use rather than prescription fill surrogates, eliminating misclassification biases common in claims datasets.

### 3.4. Documentation of Discontinuation and Reasons for Discontinuation

Information on the timepoint of the patient-reported premature treatment discontinuations in the registry was mandatorily complemented by information on the primary reason. For the present analysis, all reported discontinuations were explicitly attributed to either ADR or insufficient efficacy, dependent on patient/physician-provided information, because these reasons represent the predominant clinically meaningful drivers of early termination. ADR-related discontinuations included any drug-related adverse effects deemed intolerable or clinically significant. Insufficient efficacy-related discontinuations were defined as discontinuations due to a lack of adequate clinical benefit as judged by both patients and physicians. Examples of ADR entries included cognitive impairment, sedation, weight gain, gastrointestinal complaints, and paresthesia, whereas inefficacy entries included ‘no meaningful reduction in attacks’, no perceived benefit’, or ‘no improvement despite adequate dose’. All free-text entries were reviewed and assigned to either ADR or inefficacy.

### 3.5. Statistical Comparisons and Analytical Convention

Cumulative number (n) and proportions (percent) of ADR-related, inefficacy-related, and overall discontinuations were extracted from the registry results corresponding to six months of therapy. Based on these raw data, pairwise comparisons for the two key discontinuation parameters and the discontinuation rates were generated across the three cohorts: HEVP versus CGRP, LEVP versus CGRP, and HEVP versus LEVP. In addition, discontinuation rates were biometrically compared between (a) females vs. males and (b) EM vs. CM patients. Statistical comparisons were performed using chi-square tests, and effect estimates were reported as odds ratios (ORs) and relative risks (RRs)—both with corresponding 95% confidence intervals, representing the likelihood of treatment discontinuation (ADR-related, inefficacy-related, or overall) in the comparator cohort relative to the reference cohort. Reported effect sizes (ES) refer to the absolute risk difference between cohorts, corresponding to the inverse of the reported number needed to harm (NNH). Given the large sample size, statistical significance was interpreted in the context of effect size measures to distinguish nominal from clinically relevant differences. All inferential analyses were exploratory, and a Bonferroni correction was used to adjust for multiplicity. Missing data were rare and occurred primarily in baseline descriptors; cases with incomplete information were excluded from the respective analyses (complete-case approach). No imputation was performed, as treatment persistence and discontinuation outcomes required explicit documentation in the registry.

### 3.6. Methodological Scope Relative to Prior Work

The methodological design diverges intentionally from previous claims-based persistence studies in several key respects. First, while earlier studies inferred persistence from refill patterns, the present registry design relies on explicit physician documentation of therapy continuation or discontinuation. Second, the present analysis incorporates CGRP monoclonal antibodies, which were not available during the earlier claims analysis. Third, the current registry study includes preventive therapies with a lower evidence level, offering a broader picture of real-world prescribing patterns. Fourth, by focusing exclusively on persistence and on two primary discontinuation reasons—ADR and insufficient efficacy—the present study emphasizes a clinically meaningful and patient-centered outcome that cannot be derived from insurance claims.

This methodological framework was chosen to ensure maximal clinical relevance, internal validity of discontinuation categorization, and applicability to real-world treatment decisions in specialized care settings.

### 3.7. Ethics and Data Protection

This non-interventional, retrospective analysis was conducted in accordance with the ethical standards of the Declaration of Helsinki (latest revision, 2013) and the applicable German and European regulations governing health-care research and data protection. Because the study was based entirely on anonymized, routinely collected data from the German Pain e-Registry (GPeR), no additional ethical approval or patient contact was required.

The GPeR operates under the joint governance of the Center of Excellence for Health Care Research at the Institute of Neurological Sciences and the German Pain League. Both organizations maintain independent steering committees responsible for scientific oversight, data governance, and ethical compliance. The study concept and the planned use of anonymized registry data were reviewed and formally approved by these committees prior to analysis.

Both patients and pain centers provided written informed consent prior to enrolment in/use of the GPeR, explicitly authorizing the anonymized use of their data for research and quality assurance purposes. No personally identifiable information was available to investigators at any stage of this analysis.

All data handling and statistical processing adhered strictly to the principles of the European Union General Data Protection Regulation (EU-GDPR, Regulation EU 2016/679) and corresponding German data protection legislation. Data were stored and processed on secure, encrypted servers located within Germany. Each data record was verified for completeness and consistency prior to inclusion, and—in compliance with the EU-GDPR and the German Federal Data Protection Act—only depersonalized, non-traceable data were mirrored, and only these anonymized datasets were used for the biometric procedures described in this paper.

The study was registered prior conduct with the European Network of Centers for Pharmacoepidemiology and Pharmacovigilance (ENCePP) in the EU electronic registry of post-authorization, epidemiological and observational studies (EUPAS105823).

### 3.8. Reporting and Transparency

The conduct and reporting of this study followed the STROBE (Strengthening the Reporting of Observational Studies in Epidemiology) recommendations for observational research. All analyses were performed according to a pre-specified statistical analysis plan. In line with good research practice, the analytic workflow was documented in detail, including data cleaning, harmonization steps, and quality control procedures. No external funding influenced data selection, analysis, or interpretation. The corresponding author had full access to all data and final responsibility for submission.

### 3.9. Availability of Data and Materials

Data access is restricted and subject to registry governance approval. Aggregated or summary data may be made available upon reasonable request to the corresponding author and following authorization by the GPeR steering committee of the Center of Excellence for Health Care Research at the Institute of Neurological Sciences.

## 4. Results

The analysis included a total of 36,465 patients who provided information on their pharmacological migraine prevention to the GPeR, distributed across the three preventive therapy groups of interest: 12,347 patients receiving subcutaneous CGRP monoclonal antibodies, 23,353 patients receiving HEVP, and 765 receiving LEVP. Within the HEVP group, 7443 patients were treated with beta-blockers, 6846 with tricyclic antidepressants, 6072 with topiramate, 2453 with flunarizine, and 539 with valproic acid.

### 4.1. Population Characteristics

Table 1 summarizes the main demographic information for the patient populations evaluated. The proportion of females was consistently high (77.0%) and remarkably similar between groups, irrespective of treatment class. Mean age was likewise comparable across cohorts (on average 41.1 ± 10.5 years), as well as the average duration of migraine disease, with 12.3 ± 5.0 years. The distribution of migraine type was highly consistent across all cohorts. The average proportion of EM patients was 81.2% and ranged from 79% to 84%, whereas 18.8% presented with CM (range of 16–21%).

### 4.2. Treatment Persistence

Overall discontinuation rates within 6 months were 32.4% in females and 30.4% in males (Table 2). Although the large sample size resulted in statistically significant differences (*p* < 0.001), related effect estimates for overall treatment discontinuations were minimal: OR 0.91 (95% CI: 0.86–0.96), RR 0.97 (95% CI: 0.96–0.99), ES 0.018, and NNH 50.

Similarly, overall discontinuation rates were found for EM and CM with 31.5 and 33.6%. While statistically significant again (*p* < 0.001), the effect size was small: OR 1.10 (95% CI: 1.04–1.16), RR 1.45 (95% CI: 1.01–1.05, ES 0.017, and NNH 50.

**Figure 1:**
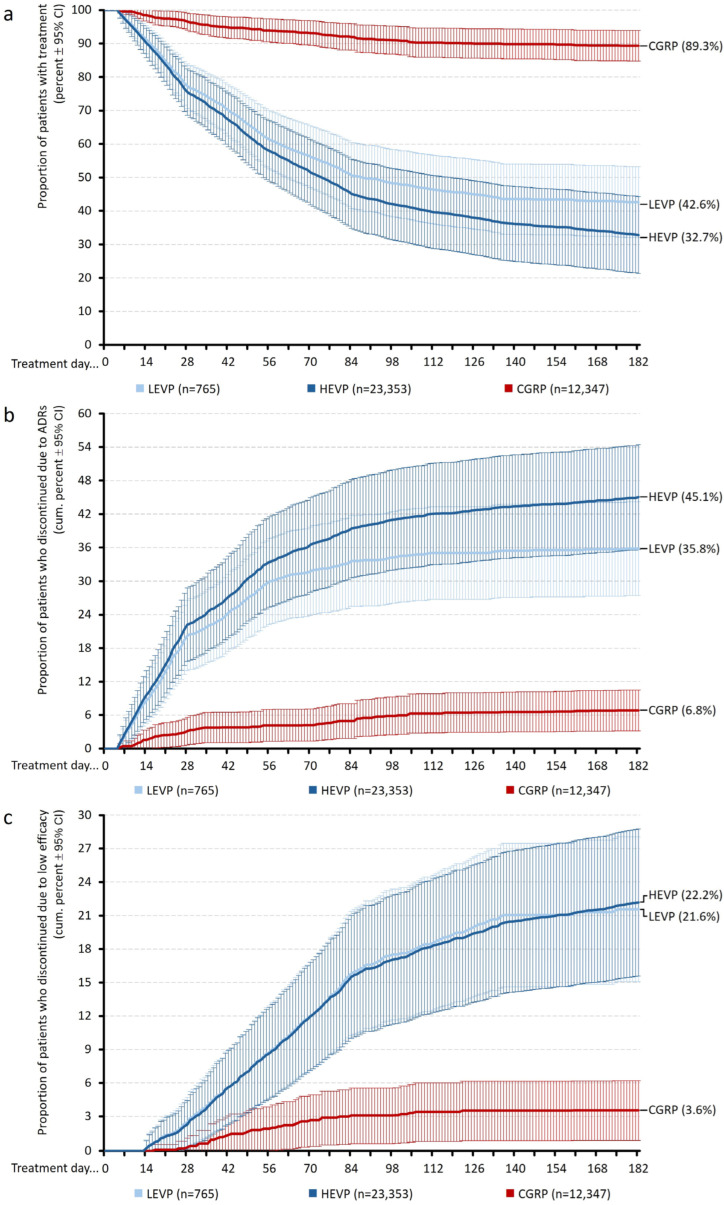
Treatment persistence and discontinuation patterns for LEVP, HEVP, and CGRP mAB over 6 months. Kaplan–Meier curves ± 95% confidence intervals (CI) showing (**a**) overall treatment persistence (upper panel), and cumulative proportion of patients discontinuing treatment due to (**b**) adverse drug reactions (ADRs, middle panel), and (**c**) low efficacy (lower panel) for traditional oral migraine preventives with low (LEVP) and high-evidence (HEVP), as well as monoclonal antibodies against calcitonin gene-related peptide (CGRP).

Kaplan–Meier curves describing treatment persistence over the 6-month evaluation period (see Figure 1a) showed clear differences between the three treatment cohorts. The persistence trajectory for the CGRP group remained consistently high throughout the whole observation period, with a continuous retention of nearly all patients for the first several weeks, followed by a very slight decline. By day 182, the persistence rate in the CGRP cohort was 89.3% (95% CI: 84.8–3.9%). In contrast, both oral preventive groups exhibited substantially lower retention. In the HEVP group, the persistence curve declined early and steadily throughout the observation period, resulting in a 182-day persistence rate of 32.7% (95% CI: 21.3–44.3%). The LEVP cohort showed an intermediate course, with an initial decline similar to but slightly less pronounced than that observed in the HEVP cohort, and a persistence rate of 42.6% (95% CI: 32.0–53.2%) at the end of month 6.

**Figure 2:**
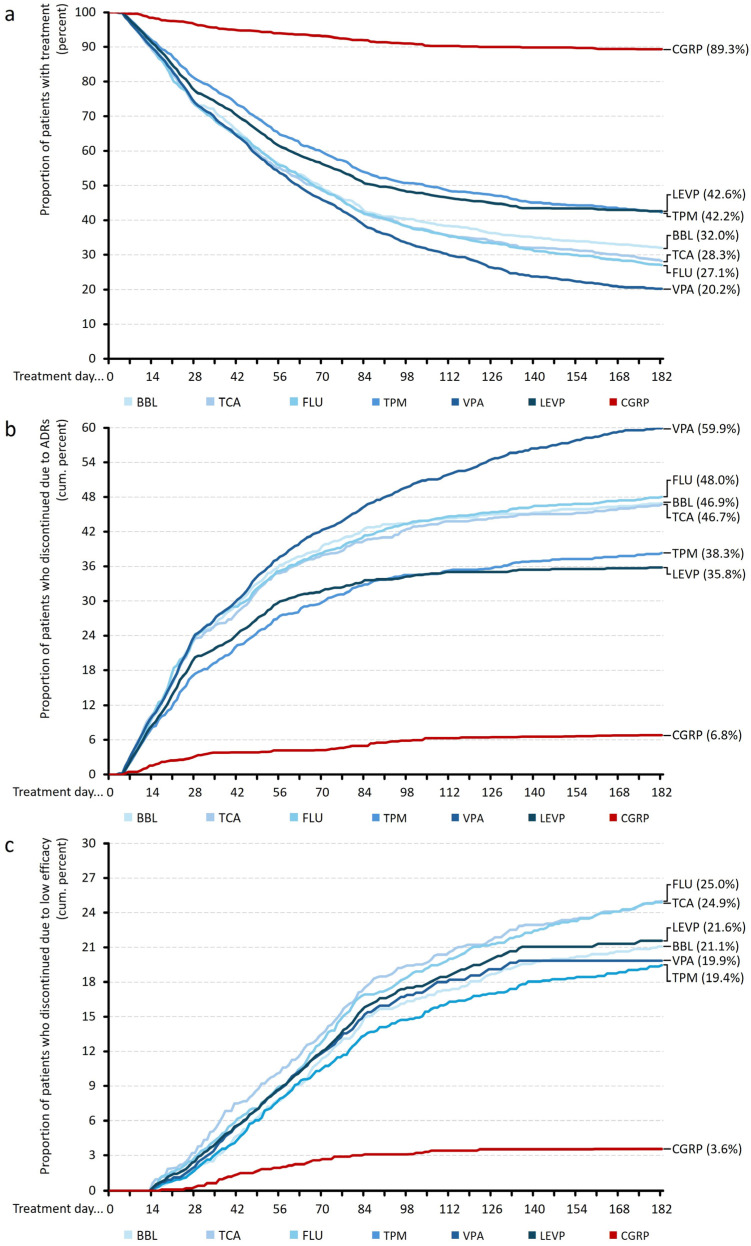
Comparative treatment persistence and discontinuation patterns across all preventive drug classes. Kaplan–Meier curves illustrating (**a**) individual and aggregated treatment persistence patterns (upper panel), as well as cumulative discontinuing slopes for (**b**) adverse drug reactions (ADRs, middle panel), and (**c**) low efficacy (lower panel). Trajectories shown are those for β-blockers (BBL), tricyclic antidepressants (TCA), fluoxetine (FLU), topiramate (TPM), and valproate (VPA), oral high- (HEVP) and low-evidence migraine preventives (LEVP), and CGRP monoclonal antibodies.

The substance-level trajectories within the HEVP group (see Figure 2a) demonstrated considerable heterogeneity. Worst treatment persistence was found for valproic acid, with a retention rate of only 20.2% at the end of month 6. Among the beta-blockers, persistence decreased continuously over the evaluation period, with a marked reduction during the first four to six weeks and a final persistence rate of 32.0%. Tricyclic antidepressants showed the steepest early decline within the HEVP group, with substantial drop-out occurring within the first month and a continuous reduction thereafter, ending up with 28.3%. Flunarizine demonstrated a comparatively less pronounced early decline than beta-blockers or tricyclic antidepressants but nonetheless showed a persistent downward trajectory across the observation window that ends with 27.1%. Topiramate exhibited the most favorable 6-month persistence pattern among the HEVP substances (42.2%), with a higher proportion of patients remaining on therapy during the first two months and a more gradual decline thereafter, although its final persistence level remained markedly lower than that of the CGRP and comparable to those of the LEVP cohort.

### 4.3. ADR-Related Discontinuations

Cumulative discontinuation due to adverse drug reactions (ADRs) differed substantially across the three treatment groups (Figure 1b). In the CGRP cohort, 6.8% (95% CI: 3.2–10.5%) of patients had discontinued therapy due to ADR by month 6. In contrast, ADR-related discontinuation occurred in 45.1% (95% CI: 35.7–54.4%) of patients receiving HEVP and in 35.8% (95% CI: 27.4–44.2%) of those receiving LEVP

Pairwise statistical comparisons (shown in Table 3) revealed marked contrasts between cohorts. When comparing HEVP with CGRP therapy, the OR for ADR-related treatment discontinuation was 11.2 (95% CI 10.4–12.0), with an RR of 1.7 (95% CI 1.67–1.72), an ES of 0.390, and an NNH of 3 (*p* < 0.001). Comparison of LEVP with CGRP therapy yielded an OR of 7.6 (95% CI 6.5–9.0), an RR of 1.45 (95% CI 1.4–1.5), an ES of 0.243, and an NNH of 3 (*p* < 0.001). In comparing HEVP to LEVP, the OR was 1.47 (95% CI 1.3–1.7), the RR was 1.2 (95% CI 1.1–1.2), the ES was 0.033, and the NNH was 11 (*p* < 0.001).

**Figure 3:**
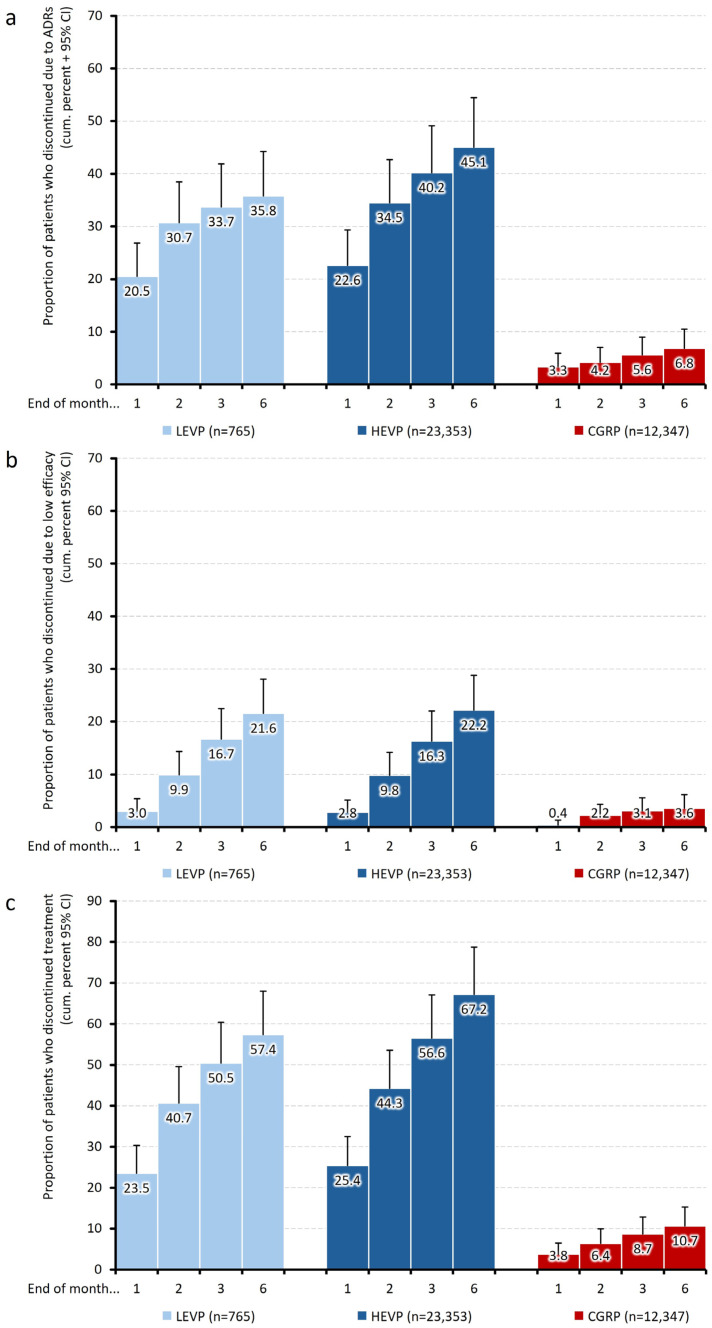
Cumulative discontinuation rates. Discontinuation rates (+95% confidence intervals, CIs) due to (**a**) adverse drug reactions (ADRs, upper panel), (**b**) inadequate/low efficacy (middle panel), and (**c**) both reasons (lower panel) at months 1, 2, 3, and 6.

Beyond the time-dependent ADR discontinuation trajectories, analyses at the ends of months 1, 2, 3, and 6 (see Figure 3a) showed that in both oral preventive groups, the majority of ADR-related discontinuations occurred within the first one to two months (85.6a% for LEVP and 76.5a% for HEVP), whereas in the CGRP cohort, ADR-driven drop-out occurred at a significantly lower and stable rate throughout the observation period.

Among HEVP, valproic acid was the migraine prophylactic with the highest ADR-related discontinuation rate (59.9% until the end of month 6), followed by flunarizine (48.0%), beta blockers (46.9%), tricyclic antidepressants (46.7%), and topiramate (with 38.3%; Figure 2b).

### 4.4. Discontinuation Due to Insufficient Efficacy

Cumulative discontinuation due to documented insufficient efficacy at six months (shown in Figure 1c and Figure 2c) was 3.6% (95% CI: 0.9–6.2%) in the CGRP cohort, 22.2% (95% CI: 15.6–28.8%) in the HEVP cohort, and 21.6% (95% CI: 15.1–28.1%) in the LEVP cohort. The pattern of early inefficacy-related drop-out showed a rapid rise within the first several weeks in both oral preventive cohorts, whereas the CGRP cohort displayed a consistently low and gradual accumulation of inefficacy-related terminations over time.

Statistical comparisons again demonstrated pronounced differences between cohorts (Table 4). When comparing HEVP with CGRP therapy, the OR for inefficacy-related treatment discontinuation was 7.7 (95% CI 7.0–8.5), with an RR of 1.24 (95% CI 1.23–1.25), an ES of 0.243, and an NNH of 5 (*p* < 0.001). The comparison of LEVP versus CGRP yielded an OR of 7.5 (95% CI 6.1–9.1), an RR of 1.22 (95% CI 1.18–1.28), an ES of 0.291, and an NNH of 6 (*p* < 0.001). In contrast, the comparison between HEVP and LEVP showed no statistically significant difference (*p* = 0.885), with an OR of 1.04 (95% CI 0.87–1.23), an RR of 1.01 (95% CI 0.97–1.05), an ES of 0.003, and an NNH of 164.

Inefficacy-related discontinuation curves over time, as well as event rates after 1, 2, 3, and 6 months (see Figure 3b), showed similar temporal patterns between HEVP and LEVP, with most discontinuations occurring within the first eight to ten weeks, whereas the CGRP curve remained nearly flat.

Among HEVPs, flunarizine (25.0%) and tricyclic antidepressants (24.9%) were the migraine prophylactics with the highest inefficacy-related discontinuation rates until the end of month 6, followed by beta blockers (21.1%), valproic acid (19.9%), and topiramate (19.4%; Figure 2c).

### 4.5. Overall Discontinuation

The cumulative overall discontinuation rate at months 1, 2, 3, and 6 increased from 3.8 to 10.7%, 66.0% in the HEVP cohort, from 23.5 to 57.4% for patients with LEVP, and from 25.4 to 67.2% with HEVP (see Figure 3c). These values reflect both causes for therapy termination, including ADR and insufficient efficacy. Monthly trajectories demonstrate a rapid and pronounced early decline in the HEVP and LEVP cohorts, beginning within the first month of therapy and continuing thereafter, while the CGRP cohort exhibited only a minimal and gradual reduction over time.

Pairwise comparisons shown in Table 5 confirmed substantial differences in overall discontinuation. The OR for overall treatment discontinuation in HEVP versus CGRP was 17.2 (95% CI 16.1–18.3), with an RR of 2.73 (95% CI 2.67–2.78), an ES of 0.539, and an NNH of 2 (*p* < 0.001). When comparing LEVP to CGRP therapy, the OR was 11.3 (95% CI 9.7–13.2), with an RR of 2.1 (95% CI 1.9–2.3), an ES of 0.322, and an NNH of 2 (*p* < 0.001). In the comparison between HEVP and LEVP, the OR was 1.5 (95% CI 1.3–1.8), the RR was 1.3 (95% CI 1.2–1.4), the ES was 0.037, and the NNH was 10 (*p* < 0.001).

Across all discontinuation categories, CGRP therapies consistently exhibited the lowest cumulative termination rates over the six-month evaluation period, whereas HEVP displayed the highest rates and the LEVP cohort showed intermediate values, with patterns more closely resembling HEVP for ADR-related and inefficacy-related discontinuations and falling between CGRP and HEVP for overall discontinuation.

## 5. Discussion

The present registry-based analysis provides an extensive real-world evaluation of treatment persistence and clinically documented reasons for discontinuation across three major categories of preventive migraine therapies—CGRP mABs, high-evidence oral preventive medications, and low-evidence preventive therapies. In doing so, it addresses several critical gaps left by earlier claims-based research, most notably the study by Hepp and colleagues [4], which has remained a central point of reference in the discussion surrounding real-world adherence to oral migraine preventives. The findings of the current study reinforce some of the earlier observations while also extending them substantially by incorporating modern CGRP biologics, documenting low-evidence therapies, and providing clinically verified discontinuation reasons that claims data cannot capture.

A central observation of this study is the markedly higher persistence with CGRP monoclonal antibodies compared with HEVP and LEVP. The CGRP cohort maintained a persistence rate of 89.3% at approximately six months, which contrasts with the 32.7/42.6% persistence observed within the HEVP/LEVP cohorts. This divergence corresponds to a fundamentally different trajectory of treatment survival: while the CGRP curve demonstrates minimal early attrition and only gradual decline, the oral preventive curves decline steeply within the first several weeks, consistent with the classic early drop-off pattern identified in earlier real-world analyses of oral migraine preventives [4]. The persistence observed for HEVP in this study aligns closely with the findings of Hepp et al. [4], who reported that approximately 25% of patients remained on oral prophylactic therapy after six months. However, the nearly 90% persistence observed for CGRP monoclonal antibodies far exceeds any preventive therapy performance described in earlier claims or observational work, suggesting a substantial shift in real-world therapeutic tolerability and acceptability.

A key advantage of registry-based data is the ability to evaluate clinically validated reasons for discontinuation. Across the comparator groups, discontinuation due to adverse drug reactions and insufficient efficacy were substantially more common among patients receiving oral preventive agents. ADR-related discontinuation occurred in 45.1% of HEVP patients and in 35.8% of LEVP patients, compared with only 6.8% in the CGRP group. These findings correspond to very large odds ratios when comparing HEVP or LEVP with CGRP monoclonal antibodies, underscoring the scale of difference in tolerability observed in real-world practice. While claims analyses could only infer tolerability from patterns of early discontinuation or treatment switching, our registry analysis demonstrates directly that ADRs are a dominant and explicit reason for terminating oral preventive therapy in clinical care.

Similar patterns emerge when comparing discontinuations due to insufficient efficacy. Despite the strong evidence base for HEVP agents in controlled trials, insufficient efficacy was cited as the reason for discontinuation in 22.2% of HEVP patients and in 21.6% of those in the LEVP cohort, compared with only 3.6% of patients receiving CGRP monoclonal antibodies. Notably, insufficient efficacy-related discontinuation occurred at practically identical rates in the HEVP and LEVP groups, which suggests that in real-world settings, the efficacy advantages of evidence-based conventional preventives may be diminished by inadequate dosing, intolerance to titration, efficiency discrepancies, or patient preferences. CGRP therapies, in contrast, demonstrated very low inefficacy-related drop-out, consistent with their robust clinical efficacy in randomized controlled trials and in other real-world studies.

Overall discontinuation rates reinforce these patterns. Two-thirds of patients in the HEVP cohort and more than half of those in the LEVP cohort discontinued their preventive therapy within six months, compared to only one in ten patients receiving CGRP biologics.

In Germany, reimbursement regulations strongly shaped treatment sequences during most of the observation period. Until April 2022, CGRP monoclonal antibodies were reimbursed only if all oral high-evidence preventives had failed or were contraindicated in episodic migraine, and if all oral high-evidence preventives plus onabotulinumtoxin A had failed or were not tolerated in chronic migraine. From April 2022 onward, access was partially eased only for erenumab in episodic migraines after at least one prior preventive failure, whereas the stricter criteria remained in place for all other CGRP monoclonal antibodies.

In this regulatory context, an additional driver of oral preventive discontinuation may have been the intention to fulfill eligibility criteria and subsequently switch to CGRP-targeted therapies. This mechanism may therefore have further reduced the apparent persistence of conventional agents beyond what would be expected based solely on tolerability or efficacy. These differences are also reflected in the extremely low NNH when comparing oral agents to biologics, emphasizing that for every two to three patients treated with HEVP or LEVP rather than CGRP antibodies, one additional patient will discontinue therapy due to ADR or insufficient efficacy.

The secondary analysis of individual HEVP substances further enriches the understanding of oral preventive performance. Tricyclic antidepressants exhibited the steepest early decline, consistent with their well-known anticholinergic and sedative side effects. Beta-blockers showed a continuous decline with substantial early attrition, which likely reflects both side-effect burden and limited perceived efficacy in some patients. Topiramate, despite being the only oral agent with strong evidence in chronic migraine trials, showed a more favorable but still limited persistence profile, flunarizine displayed intermediate behavior, and valproic acid struggles with the discrepancy of acceptable efficacy continuation rates, but unacceptable ADR-related discontinuation rates. These intra-group differences highlight how the umbrella category of “high-evidence preventive medications” contains pharmacologically heterogeneous agents whose real-world performance varies widely, a nuance that claims-based analyses have not previously been able to capture in comparable detail. In this context, it should be noted that the HEVP/LEVP classification used in this analysis reflects German guidelines and reimbursement frameworks and may differ from international recommendations. For example, evidence gradings for specific agents such as candesartan or flunarizine differ between national and international guidelines, which should be considered when generalizing these findings to other healthcare systems.

Notably, these findings align closely with the results of the open-label, randomized HER-MES trial [13], which directly compared representatives of both preventive drug classes under controlled but practice-proximal conditions. In HER-MES, early discontinuation of topiramate occurred in nearly 40% of patients—primarily due to adverse events—whereas treatment with its comparator, the CGRP receptor antagonist erenumab, was discontinued far less frequently. Although the absolute values in our cohort cannot be compared directly due to differences in design, population, and definitions of discontinuation, the direction and magnitude of the effect are consistent. The convergence between our real-world findings and this randomized trial suggests that the differences observed by us are not merely artefacts of real-world treatment heterogeneity but likely reflect inherent differences in tolerability and acceptability between the drug classes. The substantially lower rate of discontinuation with monoclonal CGRP antibodies in our data therefore supports the external validity of the HER-MES results and underscores the therapeutic advantage of a CGRP antibody-based migraine prophylaxis regarding persistence and adherence.

Differences in treatment retention between oral preventives and injectable CGRP monoclonal antibodies may partly be driven by the distinct modes of administration. Daily oral medications, such as topiramate, require continuous patient engagement and impose a higher adherence burden. Missed doses, perceived side effects, and fluctuating motivation can all accumulate over time and contribute to treatment discontinuation. In contrast, therapies administered monthly or at even longer intervals—such as CGRP monoclonal antibodies—reduce the frequency of patient–medication interaction and may consequently mitigate adherence barriers. However, the improved persistence observed with CGRP antibodies is unlikely to be explained solely by injection frequency. Even when accounting for the simplified dosing schedule, the substantially lower rate of adverse events associated with them represents an important driver of improved retention. Monthly administration may facilitate persistence, but tolerability appears to be the primary determinant of longer-term treatment continuation. All in all, the data suggest that formulation and dosing interval influence adherence, but that drug-specific tolerability profiles exert a larger effect. The interaction between these factors may explain why treatment persistence with CGRP monoclonal antibodies is consistently higher than with orally administered preventives across both real-world studies and randomized comparative trials.

Taken together, the findings of this registry-based analysis highlight the central role that tolerability and real-world effectiveness play in determining therapeutic success in migraine prevention. Even highly efficacious agents in clinical trials may exhibit poor persistence in real-world practice if side effects or insufficient benefit led to early discontinuation. CGRP monoclonal antibodies appear to overcome many of these limitations, reflecting a favorable balance of tolerability, convenience, and clinical benefit as perceived by patients and clinicians in routine care. These results also underscore the limitations of relying solely on guideline-level evidence when choosing preventive medications; although HEVP agents are supported by robust clinical trial data, their real-world acceptability remains limited.

This analysis complements and extends previous work on that issue by providing a more comprehensive overview of current preventive treatment patterns in the era of CGRP monoclonal antibodies. Whereas the Hepp study [4] demonstrated the structural problem of poor persistence with oral preventives based on prescription refill patterns, the present study confirms these findings using clinically validated documentation and shows that modern biologics substantially alter the therapeutic landscape. Furthermore, by including LEVP and substance-specific analyses within the HEVP group, the study provides a richer representation of real-world prescribing and discontinuation dynamics.

In summary, the distinct persistence patterns, combined with the markedly different rates of ADR- and inefficacy-related discontinuation, suggest that CGRP monoclonal antibodies represent a significant advancement in the preventive treatment of migraine. These results are highly relevant for clinical decision-making and guideline development, highlighting the importance of considering real-world persistence and patient-centered outcomes when selecting preventive therapies.

## 6. Strengths and Limitations

This study possesses several important strengths that enhance its relevance to contemporary migraine care, particularly in the context of evaluating real-world preventive treatment performance. A major strength lies in the use of a specialized, clinically curated registry rather than insurance claims databases. The GPeR documents clinically verified diagnoses, patient-reported measures, patient-/physician-controlled treatment decisions, adverse drug reactions, and explicit reasons for discontinuation. This stands in contrast to claims data, which infer discontinuation and cannot determine whether treatment cessation resulted from medication intolerance or absent efficacy. By relying on structured clinical documentation rather than administrative coding, the present analysis allows a more accurate representation of therapeutic retention and more precise attribution of discontinuation to adverse effects or insufficient efficacy.

Another key strength is the broad spectrum of preventive treatments examined. The study incorporates CGRP monoclonal antibodies as well as HEVP and LEVP, thereby capturing the full landscape of treatments currently used in real-world practice. Unlike the prior claims-based analysis, which predated the availability of CGRP monoclonal antibodies and did not analyze low-evidence therapies, the present registry-based approach enables direct comparison of modern biologics with the full range of oral preventives. This provides a nuanced understanding of real-world treatment survival in contemporary clinical care and extends earlier findings into the era of mechanism-based migraine prevention.

The ability to examine discontinuation across individual substances within the HEVP group constitutes another methodological advantage. Beta-blockers, tricyclic antidepressants, flunarizine, valproic acid, and topiramate demonstrate distinct real-world persistence trajectories, highlighting important intra-class variability that is obscured in analyses that treat high-evidence preventives as a homogeneous group. Such substance-level differentiation reflects the pharmacological diversity within HEVP agents and enhances the clinical specificity of the findings.

Despite these strengths, several limitations merit consideration. Foremost among these is the absence of direct clinical outcome data, such as reductions in monthly migraine days, headache severity, acute medication use, functional impairment, or other patient-reported outcomes. As a result, we were not able to determine whether the observed patterns of persistence correlate with improvements in migraine frequency or overall clinical effectiveness.

However, the absence of direct clinical outcome measures must be weighed against the methodological value of the study’s primary endpoint: treatment discontinuation. Therapy termination is a “hard” and meaningful endpoint in real-world practice. It reflects a combined judgement—made jointly by the treating physician and the patient—that the therapy is not acceptable, either due to intolerable adverse effects or because the perceived therapeutic benefit is insufficient. As such, discontinuation integrates multiple dimensions of treatment performance, including tolerability, efficacy, burden of use, and patient preference. In this sense, discontinuation can be viewed as a real-world behavioral expression of treatment success or failure—the patient’s and clinician’s “vote with their feet.” While it does not directly quantify reductions in migraine days, it does provide a robust indicator of overall therapeutic acceptability.

Another limitation is that the registry did not consistently capture starting or titrated doses of the preventive medications. Dose-related tolerability effects, particularly during the early treatment phase, may therefore have contributed to discontinuation and could not be controlled for in the present analysis.

In addition, the analysis was restricted to a six-month timeframe. Although early discontinuation is the most clinically consequential and captures the critical period during which most withdrawal decisions occur, longer-term persistence patterns remain outside the scope of this work. Additionally, the study did not stratify by migraine subtype (episodic vs. chronic) or by comorbidities, which may influence treatment trajectories. The registry population is drawn from specialized pain centers, which may result in a sample that differs from patients treated exclusively in primary care settings. Therefore, the generalizability to broader populations should be considered with appropriate caution.

Finally, although the registry provides explicit documentation of discontinuation reasons, such documentation ultimately depends on clinician judgment and may be influenced by subjective interpretation or incomplete information at the time of decision-making. Nonetheless, compared with claims-based datasets, this registry-based approach offers substantially improved clarity and validity with respect to the clinical factors driving treatment decisions.

## 7. Conclusions

In this large, registry-based evaluation of preventive migraine therapies, subcutaneous CGRP monoclonal antibodies demonstrated substantially higher real-world treatment persistence and markedly lower rates of discontinuation due to adverse drug reactions or insufficient efficacy than either HEVP or LEVP. The six-month persistence of nearly 90% in the CGRP cohort contrasts sharply with the pronounced early and sustained drop-out observed in oral preventive groups, where two-thirds of patients receiving high-evidence oral agents and more than half receiving low-evidence agents discontinued therapy within the same period. These differences were further reflected in the disproportionately high proportions of ADR- and inefficacy-related discontinuations among oral preventive users, compared with consistently low rates among biologic users.

The analysis expands upon prior claims-based evidence by evaluating contemporary treatment options, including biologics, and by documenting clinically verified reasons for therapy termination that cannot be captured in administrative datasets. The findings underscore the clinical value of CGRP-targeted therapies and suggest that real-world tolerability and sustained subjective benefit play critical roles in long-term adherence to preventive treatments. Moreover, the results highlight the limitations of conventional oral preventives in real-world practice and emphasize the importance of considering both clinical trial evidence and real-world treatment survivability when selecting therapies for migraine prevention.

## Figures and Tables

**Table 1:** Baseline characteristics of treatment cohorts.

Main Treatment Cohort Characteristics									
Treatment	BBL	TCA	FLU	TPM	VPA	LEVP	CGRP	HEVP	ALL
Cohort size (n)	7443	6846	2453	6072	539	765	12,347	23,353	36,465
Females (n)	5740	5372	1892	4715	247	594	9532	18,135	28,092
(%)	77.1	78.5	77.1	77.7	45.8	77.6	77.2	77.7	77.0
Age (years; mean)	41.2	40.6	41.0	41.7	41.3	40.6	41.0	41.2	41.1
(standard deviation)	10.5	10.4	10.5	10.7	10.4	10.5	10.5	10.5	10.5
Migraine duration (years; mean)	12.0	12.5	12.6	12.1	12.5	12.6	11.9	12.4	12.3
(standard deviation)	5.1	5.1	5.0	5.0	5.2	4.8	5.0	5.1	5.0
Episodic migraine (EM; n)	6082	5413	2059	4942	426	642	10,049	18,922	29,613
(%)	81.7	79.1	83.9	81.4	79.0	83.9	81.4	81.0	81.2
Chronic migraine (CM; n)	1361	1433	394	1130	113	123	2298	4431	6852
(%)	18.3	20.9	16.1	18.6	21.0	16.1	18.6	19.0	18.8

Demographic and clinical characteristics across individual [β-blockers (BBL), tricyclic antidepressants (TCA), fluoxetine (FLU), topiramate (TPM), and valproate (VPA)], as well as aggregated treatment cohorts [high- (HEVP), and low-evidence migraine preventives (LEVP), and monoclonal antibodies against calcitonin gene-related peptide (CGRP). Variables include cohort size (patient number), proportion of females, age at treatment onset, migraine duration, and proportion of patients with episodic (EM) versus chronic migraine (CM).

**Table 2:** Overall treatment discontinuation rates within 6 months by sex and migraine subtype.

Overall Treatment Discontinuations Until End of Month 6				
Cohort	Female	Male	EM	CM
Treatment	ALL	ALL	ALL	ALL
Cohort size (n)	28,092	8373	29,613	6852
Event rate (n)	9097	2543	9341	2299
(%)	32.4	30.4	31.5	33.6
*p*-value (chi-square for event rate comparison)	<0.001		<0.001	
Odds Ratio for discontinuation	M→F: 0.91		CM→EM: 1.10	
(95% CI)	(0.86–0.96)		(1.04–1.16)	
Relative risk for discontinuation	M→F: 0.97		CM→EM: 1.45	
(95% CI)	(0.96–0.99)		(1.01–1.05)	
Effect size (risk difference)	0.018		0.017	
NNH	50		50	

Parameters shown are cohort size, event rates, odds ratios, and relative risks—both incl. 95% confidence intervals (CIs), effect sizes (risk difference), and corresponding numbers needed to harm (NNH) comparing overall treatment discontinuations between females vs. males (left panel) and patients with episodic (EM) vs. chronic migraine (CM; right panel). *p*-values refer to chi-square tests comparing event rates between cohorts.

**Table 3:** ADR-related treatment discontinuations within 6 months across treatment cohorts.

ADR-Related Treatment Discontinuations Until End of Month 6						
Cohort/treatment	CGRP	HEVP	CGRP	LEVP	LEVP	HEVP
Cohort size (n)	12,347	23,353	12,347	765	765	23,353
Event rate (n)	845	10,521	845	274	274	10,521
(%)	6.8	45.1	6.8	35.8	35.8	45.1
*p*-value (chi-square for event rate comparison)	<0.001		<0.001		<0.001	
Odds Ratio for discontinuation	HEVP→CGRP: 11.16		LEVP→CGRP: 7.60		HEVP→LEVP: 1.47	
(95% CI)	(10.36–12.02)		(6.45–8.95)		(1.26–1.71)	
Relative risk for discontinuation	HEVP→CGRP: 1.70		LEVP→CGRP: 1.45		HEVP→LEVP: 1.17	
(95% CI)	(1.67–1.72)		(1.38–1.53)		(1.11–1.23)	
Effect size (risk difference)	0.390		0.243		0.033	
NNH	3		3		11	

Pairwise comparison of ADR-related discontinuation rates between patients treated either with monoclonal antibodies against calcitonin gene-related peptides (CGRP), oral high- (HEVP) and low-evidence migraine preventives (LEVPs). Parameters shown are cohort size, event rates, odds ratios (ORs), and relative risks (RRs)—both incl. 95% confidence intervals (CIs), effect sizes (risk difference), and corresponding numbers needed to harm (NNHs). *p*-values refer to chi-square tests comparing event rates between cohorts.

**Table 4:** Low efficacy–related treatment discontinuations within 6 months across treatment cohorts.

Low Efficacy-Related Treatment Discontinuations Until End of Month 6						
Cohort/treatment	CGRP	HEVP	CGRP	LEVP	LEVP	HEVP
Cohort size (n)	12,347	23,353	12,347	765	765	23,353
Event rate (n)	439	5179	439	165	165	5179
(%)	3.6	22.2	3.6	21.6	21.6	22.2
*p*-value (chi-square for event rate comparison)	<0.001		<0.001		0.885	
Odds Ratio for discontinuation	HEVP→CGRP: 7.73		LEVP→CGRP: 7.46		HEVP→LEVP: 1.04	
(95% CI)	(6.99–8.54)		(6.13–9.08)		(0.87–1.23)	
Relative risk for discontinuation	HEVP→CGRP: 1.24		LEVP→CGRP: 1.23		HEVP→LEVP: 1.01	
(95% CI)	(1.23–1.25)		(1.18–1.28)		(0.97–1.05)	
Effect size (risk difference)	0.243		0.291		0.003	
NNH	5		6		164	

Pairwise comparison of inefficacy-related discontinuation rates between patients treated either with monoclonal antibodies against calcitonin gene-related peptides (CGRPs), oral high- (HEVP) and low-evidence migraine preventives (LEVPs). Parameters shown are cohort size, event rates, odds ratios (ORs), and relative risks (RRs), both incl. 95% confidence intervals (CIs), effect sizes (risk difference), and corresponding numbers needed to harm (NNHs). *p*-values refer to chi-square tests comparing event rates between cohorts.

**Table 5:** Overall treatment discontinuations (all causes) within 6 months across treatment cohorts.

Overall Treatment Discontinuations Until End of Month 6						
Cohort/treatment	CGRP	HEVP	CGRP	LEVP	LEVP	HEVP
Cohort size (n)	12,347	23,353	12,347	765	765	23,353
Event rate (n)	1317	15,700	1317	439	439	15,700
(%)	10.7	67.2	10.7	57.4	57.4	67.2
*p*-value (chi-square for event rate comparison)	<0.001		<0.001		<0.001	
Odds Ratio for discontinuation	HEVP→CGRP: 17.18		LEVP→CGRP: 11.28		HEVP→LEVP: 1.52	
(95% CI)	(16.13–18.30)		(9.67–3.16)		(1.32–1.76)	
Relative risk for discontinuation	HEVP→CGRP: 2.73		LEVP→CGRP: 2.10		HEVP→LEVP: 1.30	
(95% CI)	(2.67–2.78)		(1.93–2.28)		(1.20–1.41)	
Effect size (risk difference)	0.539		0.322		0.037	
NNH	2		2		10	

Pairwise comparison of all cause-related discontinuation rates between patients treated either with monoclonal antibodies against calcitonin gene-related peptides (CGRPs), oral high- (HEVP) and low-evidence migraine preventives (LEVPs). Parameters shown are cohort size, event rates, odds ratios (ORs), and relative risks (RRs), both incl. 95% confidence intervals (CIs), effect sizes (risk difference), and corresponding numbers needed to harm (NNHs). *p*-values refer to chi-square tests comparing event rates between cohorts.

## Data Availability

Data access is restricted. Aggregated data may be made available upon reasonable request to the corresponding author and with approval by the GPeR steering committee.
